# Getting added value from using qualitative research with randomized controlled trials: a qualitative interview study

**DOI:** 10.1186/1745-6215-15-215

**Published:** 2014-06-09

**Authors:** Alicia O’Cathain, Jackie Goode, Sarah J Drabble, Kate J Thomas, Anne Rudolph, Jenny Hewison

**Affiliations:** 1 Health Services Research, Medical Care Research Unit, School of Health and Related Research (ScHARR), University of Sheffield, Regent Street, Sheffield S1 4DA, UK; 2 Visiting Research Fellow, Social Sciences Department, Loughborough University, Loughborough, Leicestershire LE11 3TU, UK; 3 Psychology of Healthcare, Leeds Institute of Health Sciences, University of Leeds, 101 Clarendon Road, Leeds LS2 9LJ, UK

**Keywords:** Qualitative research, Randomized controlled trials, Teams

## Abstract

**Background:**

Qualitative research is undertaken with randomized controlled trials of health interventions. Our aim was to explore the perceptions of researchers with experience of this endeavour to understand the added value of qualitative research to the trial in practice.

**Methods:**

A telephone semi-structured interview study with 18 researchers with experience of undertaking the trial and/or the qualitative research.

**Results:**

Interviewees described the added value of qualitative research for the trial, explaining how it solved problems at the pretrial stage, explained findings, and helped to increase the utility of the evidence generated by the trial. From the interviews, we identified three models of relationship of the qualitative research to the trial. In ‘the peripheral’ model, the trial was an opportunity to undertake qualitative research, with no intention that it would add value to the trial. In ‘the add-on’ model, the qualitative researcher understood the potential value of the qualitative research but it was viewed as a separate and complementary endeavour by the trial lead investigator and wider team. Interviewees described how this could limit the value of the qualitative research to the trial. Finally ‘the integral’ model played out in two ways. In ‘integral-in-theory’ studies, the lead investigator viewed the qualitative research as essential to the trial. However, in practice the qualitative research was under-resourced relative to the trial, potentially limiting its ability to add value to the trial. In ‘integral-in-practice’ studies, interviewees described how the qualitative research was planned from the beginning of the study, senior qualitative expertise was on the team from beginning to end, and staff and time were dedicated to the qualitative research. In these studies interviewees described the qualitative research adding value to the trial although this value was not necessarily visible beyond the original research team due to the challenges of publishing this research.

**Conclusions:**

Health researchers combining qualitative research and trials viewed this practice as strengthening evaluative research. Teams viewing the qualitative research as essential to the trial, and resourcing it in practice, may have a better chance of delivering its added value to the trial.

## Background

Qualitative research has been undertaken with randomized controlled trials in the health field for many years [1,2]. Researchers have described the contributions qualitative research can make to trials [2-6]. These include the general contribution of ‘harnessing the benefits of trials’ as well as more specific contributions around understanding how an intervention works in practice, developing appropriate recruitment and consent procedures, and helping to interpret the trial results. Researchers have described detailed examples of some of these contributions, explicitly showing the value of the qualitative research to the specific trial with which it was undertaken by challenging the feasibility and acceptability of an intervention used in a pilot trial [1] and testing and improving recruitment strategies in a feasibility trial to ensure efficient recruitment to the full trial [7]. In the 2000s the credibility of the use of qualitative research with trials was increased by the United Kingdom Medical Research Council framework for the development and evaluation of complex interventions [8-10]. This framework highlighted the utility of using a variety of methods, including qualitative research, at different phases of an evaluation. A systematic mapping review identified the range of ways in which qualitative research has been used with trials, based on 296 peer-reviewed articles published between 2008 and 2010 which reported qualitative research undertaken with trials [3]. This review identified that qualitative research has been used to address five aspects of trials: the intervention being trialed, the design and conduct of the trial, the outcomes and processes measured in the trial, the outcomes of the trial, and the health problem under study [3]. The potential value of the qualitative research included improving external validity, interpretation, ethical practice, and efficiency of the randomized controlled trial it was undertaken with.

Given the popularity of using qualitative research with trials and its potential value there is a need to reflect on how researchers practise this endeavour. Researchers have started this process by identifying the poor methodological quality of qualitative research and a lack of integration of the qualitative and trial findings [6], describing how clinical trials units can manage qualitative research [11], presenting a framework for designing process evaluations for cluster trials [12], reflecting on how more attention to the social sciences could improve process evaluations [13], and describing strategies for combining qualitative research and trials [14,15]. An important issue that has yet to be explored is how researchers with experience of combining qualitative research and trials view the value of this endeavour. A previous study seeking the views of researchers undertaking mixed-methods studies concluded that the potential of mixed-methods research was exploited when integration of the qualitative and quantitative components of a study occurred [16,17]. A lead investigator who valued integration of findings from different methods facilitated integration [16] whereas wider structural issues such as publishing norms hindered integration [17]. As part of a wider study of the use of qualitative research with trials in health [3,18] we undertook a qualitative interview study to explore researchers’ perceptions of undertaking qualitative research with trials to understand the added value of qualitative research to the trial in practice.

## Methods

We undertook a qualitative telephone interview study of researchers in the United Kingdom (UK) with experience of working on mixed-methods evaluations combining qualitative research and trials. We used telephone rather than face-to-face interviews because they are more efficient to undertake across a large geographical area and suitable for use with professionals. Ethical approval was obtained from the University of Sheffield Ethics Committee.

### The sample

We wanted to explore the views of researchers with a wide range of experience of undertaking studies combining qualitative research and trials. We used the three quantitative components of our wider study to facilitate diversity of sampling for our interviews [18]. First, we sampled from authors of articles published between 2008 and 2010 reporting qualitative research undertaken with trials [3]. Second, we sampled from researchers identified from a trials database who undertook studies which combined qualitative research and trials ongoing between 2001 and 2010. Third, we sampled from the research leads of studies on a trials database which did not report using qualitative research on the trials database but reported doing so in a survey. This approach to sampling ensured that we did not simply include researchers from studies which had successfully published the qualitative research. We focused on UK-based researchers because facilitators and barriers may differ by country and we wanted to gain a depth picture relevant to the UK. We undertook purposive sampling of both lead investigators (likely to be leading the trial) and qualitative researchers because we believed that they would offer different perspectives. We also attended to maximum variation of the sample by including researchers who had worked on studies addressing the range of uses of the qualitative research in relation to trials as identified in our wider study [3].

We used an iterative approach for selecting potential interviewees: we purposively selected ten interviewees, invited them for interview and interviewed the six that agreed; then we purposively selected another set of potential interviewees to complement the first six interviewees. We read transcripts after each interview and discussed sampling within our team. After the first 15 interviews, we paid attention to data saturation for the aim of our study. At this stage we felt that we were hearing similar issues and decided to interview another three to six researchers in order to fill gaps within our sample relating to the range of uses of qualitative research with trials. For example, we noted a gap in our sample in terms of researchers who had undertaken qualitative research to explore the measures used in a trial and approached researchers with this experience for interview.

### Interviews

The topic guide was semi-structured, focusing both on the specific study we had used to select interviewees, and on more general views based on wider experiences of doing this type of research (see Appendix). The topic guide addressed the objectives of the qualitative research within their study and how these arose, how the qualitative research was undertaken in conjunction with the trial, what made it successful (asking interviewees for their understanding of ‘success’) or how it could have been more successful, the visibility and status of the qualitative research, and decisions about reporting findings. The topic guide was designed to allow interviewees to express both positive and negative views about the use of qualitative research with trials. The interviews were conducted during 2011 by AR and lasted between 39 and 81 minutes. They were digitally recorded with permission from interviewees. Written informed consent was obtained from all interviewees.

### Analysis

Interviews were transcribed verbatim and checked for accuracy by AR. Transcripts were returned to interviewees to allow them to identify extracts they preferred were not used in verbatim quotes. For the analysis, we followed the ‘framework’ approach [19] because we had an *a priori* issue to explore in relation to our wider study - the value of qualitative research to the trial - as well as wanting to allow for emergent issues. Our team read transcripts for familiarisation (stage one of framework), developed an initial thematic framework based on the topic guide and early transcripts (stage two), and AR coded all transcripts to this thematic framework using NVivo 9 software (QSR International, Warrington, UK) (stage three). Data related to each theme were extracted (stage four) and written up by JG for discussion within the wider team. AOC and JG read the transcripts as well as the data extracts at this stage to ensure that we kept a case focus when considering the relationships between the themes (stage five). During the analysis our team discussed individual transcripts, the content of themes, connections between themes, and interpretation. While reading full transcripts at the last stage of analysis, AOC identified a typology of relationships between the qualitative research and the trial in terms of the potential to add value to the trial. She then applied this typology to the description of the study focused on each interview. Three members of our team, in addition to the researcher who had undertaken the interviews, read all the transcripts so that within-team challenges to interpretation were grounded in the full dataset.

In the findings below we present quotes to illustrate our themes. In these quotes we identify interviewees by the roles they had within the research team of the study they were selected from.

## Results

### Description of interviewees

We approached 26 researchers and interviewed the 18 who agreed. These 18 interviewees came from three sources in our wider study: they were authors of journal articles reporting qualitative research undertaken with trials (n = 9); they were applicants on studies reporting the use of qualitative research with trials which we identified on a trials database (n = 6); and they were applicants on studies which used qualitative research with trials but did not report this on a trials database, identified through a survey of researchers listed on a trials database (n = 3).

As described earlier, we undertook purposive sampling of two types of researcher working on these sampled studies: lead investigators (who were likely to be leading the trials) and qualitative researchers. Of the 18 interviewees, 11 led the trial, 7 led or undertook the qualitative research, and 2 led both the trial and qualitative research.

We offer further description of the sample so that readers can understand the characteristics of the sample. The 18 interviewees were male (n = 5) and female (n = 13). The 18 interviewees described themselves as a quantitative researcher (n = 4), qualitative researcher (n = 5), mixed-methods researcher (n = 1), lead investigator (n = 6), and both lead investigator and qualitative researcher (n = 2). Some interviewees had experience of undertaking qualitative research to address more than one aspect of trials identified in our wider study [3]: they used qualitative research to focus on the intervention (n = 11), the trial conduct (n = 6), outcomes (n = 1), measures (n = 1), and the health problem under study in the trial (n = 2). The studies we selected them from were funded from a range of sources, particularly the National Institute for Health Research and the UK Medical Research Council.

### Overview of findings

Our focus throughout our wider study was on the value of the qualitative research to the specific trial with which it was undertaken [18]. We call this the added value to the trial. The wider study was predicated on the belief that the value of the qualitative research was maximised if it impacted on the trial in some way, for example by explaining findings, refining an intervention for use in a trial, improving recruitment practices, or helping to select outcome and process measures. Although many of our interviewees shared this underlying assumption some offered alternative perspectives on the value of the qualitative research. We identify the different ways in which interviewees described the value of undertaking qualitative research before going on to explore the different models of how qualitative research was used.

### The value of the qualitative research

Interviewees described the direct utility of qualitative research for the specific trial. We devised labels for the different types of utility they described and display and explain them in Table 1. Interviewees’ views of the value of the qualitative research often focused on value for the trial and were similar to descriptions in the literature on the contribution of qualitative research to trials, including identifying problems at the feasibility or pilot stage of a trial to prevent them occurring at the full trial stage (problem solver), helping to explain the trial results (explainer), and helping research users to understand the relevance of the trial findings in different contexts (translator). Interviewees also identified a benefit for the specific trial that had not been intended when they had started their study - engaging stakeholders important to delivering the trial and thus ensuring the successful completion of the trial (engager). However, for some interviewees the value of the qualitative research lay beyond the specific trial, impacting on future trials by informing the research team’s future practice in developing and evaluating complex interventions, allowing junior researchers the opportunity to obtain academic qualifications (trainer), and offering insights into the patient experience that had the potential to improve health service delivery generally (knowledge generator).

**Table 1:** Value of using qualitative research with trials

**Role**	**Detail of role**	**Potential value**
Problem solver	Identifying problems and solutions prior to the full trial by exploring trial feasibility, informing the intervention, developing data collection instruments.	Saving the effort of undertaking trials which prove to be unfeasible, making a full trial viable by ensuring its ability to recruit or retain participants, optimising interventions so that an expensive trial is undertaken of the best intervention.
Explainer	Explaining trial results which are null, disappointing, surprising or confusing.	Offering complementary findings which supplement or modify the conclusion of the trial.
Translator	Understanding the intervention implementation and context in the full trial in order to facilitate use of the evidence in the real world.	Increasing the utility of the evidence generated by the trial for changing practice in the real world.
Engager	Securing stakeholder engagement so they have ‘buy in’ for the trial and remain enthusiastic about the trial.	Facilitating trial viability.
Trainer	Offering an opportunity for trial managers to undertake PhDs or researchers to undertake dissertations for masters.	Increasing numbers of academically qualified and experienced researchers.
	Helping trialists working in a specific field to develop understanding of a body of interventions.	Improving evaluations of interventions in the future.
Knowledge generator	Identifying issues about the experiences of patients with different health conditions.	Improving the knowledge base about how to improve the quality of services for patients with different conditions.

Some interviewees also described an unintended consequence - the potential of qualitative research to impact negatively on a trial by acting as an intervention and thus contaminating or damaging the experiment. Concern about the therapeutic effect of qualitative research existed where the interviews and observation were considered to be more intensive than the intervention under study:

‘*particularly where the intervention you’re evaluating has got a psycho-social component, you do worry a little bit about interviewer effect … a therapeutic effect which can water down the impact of the actual intervention within the trial.’* (T17, qualitative researcher).

As well as specific benefits described in Table 1, interviewees valued the flexibility of the qualitative research to emergent situations - that it could be undertaken with a specific purpose in mind but end up offering insights about other issues. They described the need for qualitative research to evolve over time within a study rather than be constrained by the original research design, for example, in order to address difficulties that arose within the wider study. This flexibility and responsiveness was discussed as a strength and as quite different from qualitative research that was unplanned or not thought through. This flexibility was seen as complementary to the heavily protocolled approach taken to trials.

### Three models of the relationship of the qualitative research to the trial

Within our sample we identified three models of the relationship of the qualitative research to the trial based on how interviewees described their studies. The first model we call ‘the peripheral’, where the intention was not to add value to the trial but to attain a value unrelated to the trial. For example, where the trial offered an opportunity to provide a higher degree for a researcher (‘trainer’ in Table 1), or to explore an area of interest to a qualitative researcher to facilitate understanding of patients’ experiences of a disease (‘knowledge generator’ in Table 1). For some of the 18 studies that interviewees discussed in detail, a number of qualitative components were undertaken. We categorised at least one qualitative component of three of these 18 studies as undertaken within a ‘peripheral’ model.

The second model we call ‘the add-on’, where the qualitative researcher believed in the value of the qualitative research to the trial but perceived that this belief was not shared by the lead investigator of the study or key team members. They described how the team viewed the trial as the central study component, with the qualitative research as an interesting but ultimately separate activity. The intention of the lead investigator or wider team was described by interviewees as not to add value to the trial but to generate knowledge that was complementary to the trial:

*‘more quantitatively oriented trialists […] might be interested in the qualitative results in their own right, but I don’t know how interested they’re going to be in thinking about what it tells us about the trial. […] They provide a path of least resistance, if you want.’* (T11, qualitative researcher).

We categorized at least one qualitative component of four studies as ‘add-on’.

The third model was where the lead investigator viewed the qualitative research as integral, that is, essential to the evaluation because of uncertainties around the trial, the intervention, the outcome they wished to affect, or the patient group receiving the intervention. Indeed the term integral was used commonly by interviewees. These lead investigators described being driven by the need to undertake research which was applicable to the complex world in which health and healthcare operates and they could not conceive of undertaking a trial of the types of issues they were interested in without also using qualitative research. However, the practice of the study did not always match this intention. We categorised 14 qualitative components from our 18 studies as integral. There were two subgroups of this model: ‘integral in theory’ (7 qualitative components) and ‘integral-in-practice’ (7 qualitative components). We categorized studies as ‘integral in theory’ when interviewees described some added value of the qualitative research to the trial but that it was limited because the qualitative research was under-resourced, there was a lack of integration between the qualitative research and the trial:

‘*it can be used in much more creative ways than it is being I think, or it has been doing, because I think there’s still this idea that it’s just something on its own whereas it can be integrated.*’ (T1, lead investigator),

or the qualitative research was perceived as poor quality in terms of a lack of conceptual thinking or ‘intellectual intensity’:

*‘I think one of the problems that we face with qualitative research is that it is talked up a lot, but quite often the product that is delivered isn’t nearly as good as the aspirations for the discipline […] I would say that most of it falls very far short of this sort of conceptual framework, real contribution to understanding that we think it will be, […] and you come out the other end and you think, ‘I don’t know if I’ve learnt anything from this’. And when you meet really good qualitative research, you know what […] we can achieve with it, but so often, I think, the intellectual intensity that’s required to do it well isn’t applied*.’ (T8, lead investigator).

We categorized studies as ‘integral-in-practice’ when interviewees described the qualitative research impacting on the trial to the satisfaction of the interviewee by changing the outcome measures to be used or explaining the trial findings.

### Issues important to ‘integral-in-practice’ qualitative research

Where we categorised the qualitative research as ‘integral-in-practice’, interviewees described resources which maximised the potential added value of qualitative research to the trial. These resources included senior qualitative expertise on the team from beginning to end, and staff and time dedicated to the qualitative research.

#### Qualitative researcher as ‘full’ team member from the start

Both the quality of the qualitative research and its integration with the trial could be affected by who was in the team from planning through to completion of the study. Where qualitative research was integral-in-practice the qualitative researcher was the principal investigator, a joint principal investigator with a quantitative colleague, or a co-applicant. The qualitative research was designed in the original study proposal with senior qualitative expertise on the team from the outset. This contrasted with the ‘add-on’ and ‘integral-in-theory’ models where the qualitative researcher could be junior and/or have been brought into the team once the trial had started. They might have access to a senior qualitative researcher in theory but in practice this person was too busy to offer them supervision. One interviewee had been the ‘added on’ junior team member in the past:

*‘I was brought in to help finish off those interviews and then I led the focus groups […]. Because I was brought in at a late stage, and I basically came in, finished off a bit of a job and then wrote the sections for the report, but didn’t really bring it together, I’m not sure how the links were made between the two’* (T13, qualitative researcher).

On another study this interviewee felt themselves to be a ‘full’ team member from the beginning, which they felt enhanced the quality of research produced by ensuring that a better research proposal was written.

Where we categorised the qualitative research as ‘integral-in-practice’ the lead investigator had mixed-methods expertise or there was joint leadership between the quantitative and qualitative experts:

*‘I was the PI and I’m a qualitative researcher, so it’s quite unusual in that most complex intervention trials are designed by the quantitative person and the qualitative researcher is sort of bought in.’* (T9, lead investigator and qualitative researcher).

This shared expertise at the top of the team was not necessary to ensure quality qualitative research and integration with the trial but the presence of senior qualitative researchers was reported as ensuring good quality qualitative research by facilitating depth analysis and write-up. This was described in contrast to an ‘add-on’ model seen in grant applications:

*‘… there certainly are an awful lot of grant applications I see where it is quite clear that the little bit on process evaluation is just there as a bit of a token, maybe got a junior qualitative Research Assistant from a sociology department down the road to write a paragraph to go in the bid and then they cost it in for a few hours a week for the duration of the trial and clearly they are not an integral and powerful part of the study team’* (T18, lead investigator).

#### Time to undertake the qualitative research

Our ‘integral-in-practice’ studies had senior qualitative researchers as applicants with enough of their time funded to ensure good quality research was undertaken, as well as qualitative researchers to undertake the research rather than trial managers doing it alongside running the trial. This contrasted with ‘integral-in-theory’ and ‘add-on’ models where qualitative components were described as under resourced:

*‘That’s the ideal situation - where the work is properly funded’* (T3, qualitative researcher).

Time, as well as staff, was an important resource, with enough time allocated to allow for an in-depth qualitative analysis:

*‘…. allowing adequate time for analysis of qualitative data is important because it does take much longer than analysing quantitative so therefore there is a cost implication, so that’s the only thing that could be challenging when working with people who are from a more quantitative background is helping them understand the process of qualitative analysis and the time that it takes.’* (T17, qualitative researcher).

A negative case concerning the importance of resourcing the qualitative research was an interviewee describing a study which we categorized as an ‘integral-in-practice’ study as having had little money available for the qualitative research. However, the trial was also described as under-resourced. That is, there was not a disparity of resource with perceptions of under-resourced qualitative research and an adequately resourced trial. It was also the case that within the study they described there was senior qualitative research commitment and time invested in delivering the qualitative research in depth regardless of the funding available.

#### Investing time to communicate

In addition to the way teams were structured, the way they were described as operating on a day-to-day basis and how members communicated with each other was seen to contribute to the way qualitative research could add value to the trial. ‘Integral-in-theory’ and ‘integral-in-practice’ studies had integrative team practices. Where the qualitative research was an add-on, team closeness could develop over time, for example, as a colleague who was described as not ‘overly impressed’ with the qualitative research became more interested once the qualitative findings were reported (T10, qualitative researcher). Engaging the whole team and working in a collaborative way was described as facilitative to integrating different parts of the study. The use of meetings could encourage communication and reciprocal appreciation of the different work streams in the study:

*‘… the main thing was openness of communication, fostering an environment that everybody's views counts and everybody’s methodology is on the same level, and about each member of the team facilitating qualitative or quantitative research to be done, at the time that it was required, so our team meetings were very open, we all knew we didn’t know everything, so we were very open about asking questions from each other, because none of us could cover the full spectrum that everybody else had, and, keeping those channels of communication open was seen to be very good.’* (T4, lead investigator).

### The wider research environment can limit value

Interviewees discussed the wider research environment in which these mixed-methods evaluations were undertaken. Aspects of this wider environment could explain why qualitative research appeared to be peripheral, an add-on, or integral in theory. The wider environment could also affect ‘integral-in-practice’ studies in that the value of the qualitative research to the trial was not necessarily visible beyond the original research team because it was not reported in journal publications.

#### The drive to pursue academic careers

A qualitative researcher reflected on how trials could be vehicles for the career progression of some researchers:

*‘They are not driven to find a treatment that is acceptable to patients, are they? They are essentially driven by the task of undertaking a highbrow randomized controlled trial, getting publications on it and moving on to the next big thing’* (T10, qualitative researcher).

Even researchers who spoke with passion about producing research evidence for use in the real world were driven by the need to be seen within their own institutions as someone doing ‘valuable research’. Valuable research was defined as producing articles that were published in high impact journals and if the qualitative research could not produce these then it was viewed as less valuable, or even worthless, within academia:

*‘… if it’s not a three-star paper, which they define as something like obviously high impact and is top 10*% *of Web of Science categories, then it doesn’t count for anything at all, and you’re literally in their eyes wasting your time writing it. So, it’s difficult for me to justify the time writing up qualitative papers for minor journals when I could be writing high impact papers or getting more grant money in.’* (T5, lead investigator).

Pursuit of an academic career also included the need to bring in more funding and move on to the next project, resulting in teams breaking up before ‘non-priority’ papers were written, where the qualitative papers were described as the non-priority ones:

*‘… our priority now, at the moment, is just to get the trial paper published. […] I’m being honest here, there is an issue of will here. Because we have learnt a lot by doing this, including by doing the qualitative research and we’ve moved on to new projects, and we’ve just started another massive project, and so the question is where is the appetite when we’ve moved on. Who in our team is going to develop the qualitative publications from the work that we did before?’* (T8, lead investigator).

#### The value of applied qualitative research

The disciplinary background of team members and the associated research paradigms were described as significant for the value placed on the qualitative research. Some clinical specialties were described by interviewees as more sympathetic to qualitative research than others - palliative care, public health, and primary care - where one might argue the complexity of interventions is more obvious than in other specialties. A lack of expertise in qualitative research by the quantitative researchers was described as not only affecting the quality of the qualitative research by imposing quantitative ideals on it (for example wanting large numbers of interviews), but could also affect the confidence quantitative researchers had in the methodological quality of the qualitative research and therefore their willingness to consider its interaction with the trial. These differences were perceived to be due to a lack of understanding of qualitative research by quantitative researchers in the team, or even a lack of understanding that there was anything to understand. The effect of not having expertise, or constraining that expertise, on the quality of the qualitative research was not necessarily evident until it was too late, as in a case where at transcription stage it became apparent that the reality of the ‘in-depth’ interviews was that the participants *‘*only got the choice of saying ‘yes’ or ‘no” (T15, lead investigator). Additionally, some qualitative and mixed-methods interviewees described how qualitative research, applied research, or applied qualitative research might not be valued within their discipline, making it difficult to publish in a high impact journal within their discipline or a ‘decent qualitative journal’ (T5, lead investigator).

#### Funding agencies

Interviewees noted that qualitative and mixed-methods researchers were now on research commissioning panels which they perceived as creating a more favourable condition for obtaining funding for this type of work. The UK MRC framework for the evaluation of complex interventions was identified as shaping the environment within which researchers worked, promoting the value of qualitative research to funding agencies to the point that they would sometimes request that qualitative research was included within a bid:

*‘I think the commissioning process has really changed quite significantly since this was set up […] now the MRC has its famous complex trials framework. And that’s just been part of the general change across the whole community with the interdisciplinary understanding of applied health researchers coming to bear on the broader community to show that qualitative research is important, has important roles to play in trials … And so the commissions have improved dramatically.’* (T2, lead investigator).

However the availability of funds clearly exercised some interviewees. One interviewee described the funder’s view of the qualitative research as integral to the trial and their willingness to fund it fully as a key contributing factor to maximising its value. They then went on to say that *‘*to get a donor who’s willing to put the extra money in is exceptional’ (T15, lead investigator), and indeed another interviewee regarded having dedicated staff to undertake the qualitative research as *‘*quite a luxury’ (T3, qualitative researcher). It was not clear to what extent the funding agencies were contributing actively to this perceived lack of funding for fully resourced qualitative research and to what extent lead applicants were contributing to it by trying to keep the costs of a bid down, either by ‘second-guessing’ their chances of securing a large enough grant to fund the qualitative research properly or by trading on the goodwill of qualitative researchers to ‘squeeze it in’ without proper funding:

*‘because it wasn’t that the funder said you cannot have this money, but we underestimated in order to keep the costs of the study down, and hence didn’t have the time or the resource in terms of the researcher to gather the data and to analyse the data as fully as you would have liked. So I suppose that’s the take-home message really [laugh], […] we shouldn’t have been quite so ambitious in the original proposal and should have tried to seek more funding’* (T12, lead investigator).

A related issue for some interviewees was the space on the funding application form being used to give details about the trial and little or no detail about the qualitative research, an issue we also identified in our wider study [20]. Interviewees suggested that funders could help to promote the importance of describing the qualitative research by changing application forms to offer explicit space for the qualitative research:

*‘the quantitative side tends to take predominance very often in terms of the application […] and the qualitative side is much often less fully described, because there’s less space left on the application form*’ (T12, lead investigator)

to ensure it was planned in detail:

*‘As a grant reviewer, you tend to get very tired of people saying ‘Oh yes, we’ll do a mixed-methods evaluation, including a process evaluation, because it’s very important, blah blah blah’ and then they say absolutely nothing about what exactly they’re going to do […] there are very few applications you get where they can say a lot because of the fact that the forms aren’t helpful to support that.*’ (T18, lead investigator).

#### Publishing the added value

Regardless of the degree of integration of the qualitative research and the trial during the study, and its perceived value by all team members during the trial, communication of learning from this integration at the publication stage seemed to present a final challenge that proved insurmountable for some of our interviewees. They described fragmentation of teams that could leave the lower priority paper unpublished; the production of the findings of the qualitative and the quantitative research could occur at different times resulting in the communication of the value of the qualitative research in terms of explaining the trial findings being lost; or two separate sets of journal articles could be published to report the quantitative and qualitative findings separately without full attention to communicating the value of the qualitative research to the trial:

*‘… ideally we would have put more stuff from the process evaluation in the main trial paper but we didn’t … because of the word length, you know, word count restriction meant that just by reporting all the usual stuff for the primary and secondary outcome analyses, there wasn’t really room for much else.’* (T18, lead investigator).

### An improving climate

Some interviewees who had been engaged in combining trials and qualitative research for many years felt that the external climate for the endeavour had become more facilitative. This progress was in part attributed to changing priorities in healthcare research, in which trials were being used for more complex or behavioural interventions. Nonetheless, these experienced researchers wanted to see further shifts in the external research environment to maximise the value of qualitative research with trials.

## Discussion

### Summary of findings

The value of undertaking qualitative research with trials was considered to be high by researchers in our sample. Our interviewees had experience of this endeavor, either as trialists or qualitative researchers. Both types of researcher described the value of qualitative research to the specific trial with which it was undertaken in ways that have been documented in the literature: it improved the intervention and the trial conduct at the pretrial stage, explained trial findings, and illuminated issues important to the application of evidence in the real world. They also identified how the qualitative research had value other than for the trial. We identified three models of relationship of the qualitative research to the trial. In the first model ‘the peripheral’, the purpose of the qualitative research was not to have value for the trial. The trial was an opportunity to undertake qualitative research with another purpose such as to gain a research degree for a researcher. In the second model ‘the add-on’, the qualitative researcher understood the value of the qualitative research but described the study lead and the wider team viewing it as a separate complementary endeavour. Within this model there was little commitment or plan for integration, that is, that the qualitative research would have an impact on the trial. In the third model ‘integral’, there were two subgroups. For ‘integral-in-theory’ studies, the study lead viewed the qualitative research as essential to the trial due to complexities and uncertainties about the trial or intervention. However, they described how in practice the qualitative research was under-resourced in terms of senior expertise to lead it, experienced researchers to undertake it, and time to deliver it. This could lead to underdeveloped qualitative research, limiting its ability to add value to the trial. For studies we categorized as ‘integral-in-practice’, the qualitative research was described as planned from the beginning of the study, with senior qualitative expertise on the team from beginning to end, and resourced in terms of time and money. The intention in the subgroup was that the qualitative research would impact on the trial by developing the intervention, helping to select the outcome measures, or explaining the findings. The wider academic environment was described as supporting the randomized controlled trial as the most valued component of mixed methods evaluations, contributing to the occurrence of ‘add-on’ and ‘integral-in-theory’ models and the lack of visibility of the impact of the qualitative research on the trial beyond the original research team.

### Putting findings in the context of our wider study

This interview study was one component of a mixed-methods study [18]. In our wider study we found a range of ways that qualitative research had been used with trials and the potential value for the specific trial [3]. In our wider study we had to draw conclusions ourselves about the potential value of the qualitative research to the trial because researchers did not necessarily report explicitly the implications of the qualitative research for the trial [3]. Our interview study reported here explains why researchers may find it difficult to articulate this value in publications. We also found that qualitative research could be poorly described in research proposals, an issue echoed here in our interview study. We offer guidance elsewhere for writing research proposals for qualitative research with trials [20].

### Putting findings in the context of other research

The findings of this interview study are supported by other research in the health field, both specific to the use of qualitative research with trials and about mixed-methods research more generally. A review of studies combining trials and qualitative research found a lack of integration of qualitative and trial findings in study publications [6], and our systematic review of articles reporting the qualitative research undertaken with trials largely identified lessons for future trials rather than the trials they were undertaken with [3]. The interview study we report here complemented both of these reviews by identifying explanations for this lack of visible integration at the publication stage of an evaluation. A similar interview study with researchers from mixed-methods studies showed that they valued mixed-methods research and saw integration of the qualitative and quantitative findings as a mark of quality but tended not to engage in integration because of dysfunctional or multidisciplinary team working [16] and structural constraints such as publication norms [17]. Our findings are also similar to issues raised in the discussion section of a methodological article about the use of mixed-methods in palliative care where the authors identified problems such as under-resourced studies resulting in poor quality research, and difficulties in publishing integrated findings, as well as some issues not identified here such as a lack of skill mix and experience in mixed-methods research [5]. Another article described similar issues of how publishing the trial took precedence, and the inability to show the impact of the qualitative research on the trial because the qualitative research was not complete at the time of publishing the trial [21].

### Strengths, limitations, transferability, and reflexivity

The strength of the study is that it is grounded in the views of researchers who have experienced combining qualitative research and trials. The sample was taken from the quantitative component of our wider mixed-methods study [18] to ensure we had the views of researchers currently undertaking this endeavour, researchers who had seen projects through to completion, and researchers who had worked on projects where the qualitative research was likely not to be valued (not visible on a trials database). Our sample consisted largely of researchers who were successful in terms of having published the qualitative research from the study we selected them from. A limitation is that eight researchers we approached did not respond to our request for an interview or declined to be interviewed. We do not have information on why people did not take part but we need to consider the possibility that these researchers might hold different views from those expressed in our sample. One of the eight had left academia and their organisation did not pass on contact details to us. Three of the eight were identified from the survey of researchers on a trials database where the trial in the database did not mention qualitative research but the survey respondent identified that qualitative research had been undertaken; we only had three interviewees from this source in our sample and would have preferred to have more. It is possible that these researchers were too busy to commit to an interview, or that they perceived combining qualitative research and trials to be of little or no value, we simply do not know. Another possible limitation is that four interviewees took the opportunity to change or exclude parts of their transcripts from the study. Two of these interviewees excluded small extracts from being used as quotes for fear of being identified; two excluded small extracts related to descriptions of studies other than the study we had interviewed them about; and one corrected a factual answer they had given to a question. We do not believe that any of this affected the findings. A final possible limitation was that the telephone interviews might have affected our ability to develop a rapport with interviewees. We reflected on this and felt that in practice interviewees seemed to discuss their experiences with ease and in depth, ensuring we had rich and informative data for our study.

The sample was drawn from the UK where the use of qualitative research with trials is acceptable to significant funding bodies such as the National Institute of Health Research and the Medical Research Council. Researchers from other countries will have to consider whether this is the case in their specific context when thinking about the transferability of these findings.

The interview topic guide and the analysis and interpretation were shaped by our team’s belief that qualitative research undertaken with a specific trial should have value for that trial. However, we attempted to engage in open enquiry (see our topic guide) and were open to the possibility that researchers would disagree with our position. Indeed our interviewees offered definitions of the value of using qualitative research with trials where the value was to the researcher or understanding health rather than to the trial, and concerns about the qualitative research damaging the trial. Some members of our team have undertaken trials themselves and are supportive of trials for generating evidence of effectiveness. We are critical of how some trials have been undertaken, including our own research, but do not challenge the need for trials. Again, this stance shaped our research question and may have affected our sampling, analysis, and interpretation. To balance this, three members of our team had not undertaken trials or qualitative research with trials; one of these researchers undertook the interviews and one wrote the first draft of the analysis.

### Implications

Our sample of experienced researchers was generally positive about the value of qualitative research undertaken with trials, showing support for the utility of this endeavor amongst trialists as well as qualitative researchers. If researchers undertake qualitative research with a trial then we recommend that they are explicit about their reason for doing so; this will help to establish realistic expectations of the work. If researchers intend the qualitative research to be of added value to the trial then we recommend that they plan how this will occur and ensure that there are resources in terms of senior expertise, time, and qualified researchers to do this because this may help to maximise its value. The wider research environment can affect how this research is undertaken and reported. We recommend that journal editors publish papers reporting qualitative research which, for example, explain why an intervention did or did not work, as much as they publish trials which show the size of effect of an intervention in a particular context.

## Conclusions

Experienced researchers value using qualitative research with trials to address problems such as poor recruitment, a lack of understanding of why interventions work or not, or a lack of use of evidence generated by trials in practice. This value does not always occur though, because the qualitative research is not always valued in practice - indicated by it being under-resourced. A further reason is that the impact of the qualitative research on the trial occurs within some studies in ways that are invisible beyond the original research team due to structural constraints such as publishing norms, limiting its value to other researchers working on similar interventions or in similar environments. We would like to see researchers, funding agencies, universities, and journal editors place more value on articulating the impact of qualitative research undertaken with trials in order to reap the considerable benefits of this endeavour. Teams viewing the qualitative research as essential to the trial, and resourcing it in practice, can facilitate the delivery of the value of using qualitative research with trials.

## Appendix

### QUART draft interview topic guide

#### Introduction

Thank the interviewee for agreeing to the interview and to the recording of the interview. Check consent and if it is OK to record the interviews. Ask them if they have any questions before the interview begins.

Say that you are here to talk to them because of their involvement in a trial with qualitative research: [**STUDY A**].

### Interview topics to be covered during the interview

1. The interviewee’s role in the trial (PI or qualitative researcher)

2. The qualitative components of their trial and the interviewee’s perceptions of the objectives for undertaking each component if more than one (Prompt: Pick up on any elements of the trials that we have not yet identified and explore in more depth)

3. The reasons why qualitative research was undertaken with this trial

4. The rationale for qualitative research addressing the objectives above

5. The success of the qualitative research with this trial from the interviewee’s point of view (explore their meaning of ‘successful’)

6. The interviewee’s perceptions of the impact of qualitative research on the trial itself (Prompts: Did it do the right ‘work’, status of qualitative components, visibility of qualitative components, reporting of qualitative components, worth having a qualitative component)

7. Challenges and opportunities for the successful use of qualitative research in the interviewee’s study (Prompt for unintended consequences, both positive and negative)

8. Challenges and opportunities for the successful use of qualitative research in trials more generally

9. Issues related to the commissioning of the qualitative research for the trial and more generally

10. The interviewee’s perceptions of qualitative research in general

### Ending the interview

11. Is there anything else you would like to say?

12. What is the message you would like me to really take away today?

13. What would you like to see coming out of our study?

14. Close the interview and thank the interviewee for their participation.

## Abbreviations

MRC: Medical Research Council; UK: United Kingdom.

## Competing interests

The authors declare that they have no competing interests.

## Authors’ contributions

AOC and KJT designed the study. AOC, KJT and JH obtained funding. AR collected the data. JG, AOC and SJD analyzed the data. All authors contributed to interpretation of the findings. AOC wrote the first draft of the article and all authors contributed to editing the article. AOC acts as guarantor of the article. All authors read and approved the final manuscript.
